# Workload and clinical impact of MRI-based extension of reperfusion therapy time window in acute ischaemic stroke—a prospective single-centre study

**DOI:** 10.1007/s11357-025-01549-1

**Published:** 2025-02-06

**Authors:** Tímea Tünde Takács, Rita Magyar-Stang, Szabolcs Szatmári, Ildikó Sipos, Katalin Saftics, Ádám József Berki, Sándor Évin, Dániel Bereczki, Csaba Varga, Nóra Nyilas, István Bíró, Péter Barsi, Máté Magyar, Pál Maurovich-Horvat, Péter Pál Böjti, Máté Pásztor, István Szikora, Sándor Nardai, Bence Gunda

**Affiliations:** 1 Department of Neurology, Semmelweis University, Budapest, Hungary; 2 Schools of PhD Studies, Doctoral School of Neurosciences “János Szentágothai”, Semmelweis University, Budapest, Hungary; 3 Department of Emergency Medicine, Semmelweis University, Budapest, Hungary; 4 Department of Neuroradiology, Medical Imaging Centre, Semmelweis University, Budapest, Hungary; 5 Department of Neurosurgery and Neurointervention, Semmelweis University, Budapest, Hungary; 6 HUN-REN-SU Neuroepidemiological Research Group, Budapest, Hungary

**Keywords:** Ischaemic stroke, Thrombolysis, Thrombectomy, Extended time window, MRI, Real-world data

## Abstract

Current European Stroke Organisation (ESO) guidelines recommend extended time window reperfusion therapies (4.5–9 h for thrombolysis, 6–24 h for thrombectomy) based on advanced imaging. However, the workload and clinical benefit of this strategy on a population basis are not known. To determine the caseload, treatment rates, and outcomes in the extended as compared to the standard time windows. All consecutive ischaemic stroke patients within 24 h of last known well between 1st March 2021 and 28th February 2022 were included in a prospective single-centre study. Treatment eligibility in the extended time windows or wake-up strokes recognized within 4 h was based on current ESO guideline criteria using MRI DWI-PWI or DWI-FLAIR mismatch. MRI was only available during working hours (8–20 h); otherwise, CT/CTA was used. Clinical outcome in treated patients was assessed at three months. Among the 777 admitted patients, 252 (32.4%) had MRI. The thrombolysis rate was 119/304 (39.1%) in standard and 14/231 (6.1%) in the extended time window. The thrombectomy rate was 34/386 (8.8%) in standard and 15/391 (3.8%) in the extended time window. Independent clinical outcomes (mRS ≤ 2) were not statistically different in early and late-treated patients both for thrombolysis (48% vs. 28.6%, *p* = 0.25) and thrombectomy (38.4% vs. 33.3%, *p* = 0.99). Even with a limited availability of advanced imaging extending therapeutic time windows resulted in an 11.7% increase in thrombolysis and a 44% increase in thrombectomy with comparable clinical outcomes in early and late-treated patients at the price of a twofold burden in clinical and advanced imaging screening.

## Introduction

Acute ischemic stroke (AIS) management is based on acute reperfusion therapies including intravenous thrombolysis (IVT) and endovascular therapy (EVT). The European Stroke Organization (ESO) guidelines changed in 2021, resulting in the extension of the IVT time window (TW) with recombinant tissue plasminogen activator (rtPA) from 4.5 to 9 h and EVT TW for patients with large artery occlusion from 6 h up to 24 h from symptom onset in selected patients [1, 2].

The extension of treatment TW requires multimodal imaging with computed tomography (CT) perfusion (CTP) or magnetic resonance imaging (MRI) perfusion (MRP) to identify the penumbra, the salvageable brain tissue volume [3]. Based on advanced imaging, fast and slow progressors can be differentiated, and patients with a substantial amount of reversible ischaemic brain tissue without extensive infarct core can be selected for late treatment beyond the standard TWs [4, 5].

While the positive effects of treatment in extended TWs on functional outcomes are well documented in clinical trials, real-life clinical implications of broadening the range of screened patients with advanced imaging have only scarcely been reported [6, 7].

We aim to provide insight into the caseload, treatment rates, and outcomes in the extended as compared to the standard TW for thrombolysis and thrombectomy in a high-volume Stroke Centre using MRI for advanced imaging.

## Materials and methods

We prospectively collected the data of every consecutive AIS or transient ischemic attack (TIA) patient who presented within 24 h from symptom onset at Semmelweis University Stroke Centre between 1st March 2021 and 28th February 2022. All patients were initially admitted to the emergency room (ER) where board-certified neurologists performed the neurological examination. The first-choice imaging in all suspected stroke patients arriving during working hours between 08:00 and 20:00 was multimodal MRI. In the remaining hours (20:00–08:00) and if MRI was contraindicated or not tolerated by the patient, CT and CT Angiography (CTA) were performed without perfusion imaging. Thus, if a patient was unsuitable for MRI, extended TW reperfusion therapy could not be indicated. The CT scanner (64-slice Philips Incisive) is located within the ER, whereas MRI scanning (on a Philips Ingenia 1,5T scanner) is only available on another floor of the same building requiring longer patient transportation and elevator use. The standard MRI sequences in patients within 4.5 h were DWI, FLAIR, GRE T2*, PWI, and contrast-enhanced MRA; the acquisition time was 8 min. Using SWI instead of GRE T2* and additional postcontrast sagittal T1 in the late TW patients, the acquisition time was 12 min. Postprocessing to calculate core/penumbra volumes was done on the Philips IntelliSpace platform; the results were usually available within 10 min after scanning. The infarct core was defined as ADC < 620 mm2/s lesion volume and critically hypoperfused tissue (ischaemia) was defined as Tmax > 6 s volume.

All reperfusion treatment eligibility criteria were based on current ESO guidelines.

Radiological criteria for extended TW reperfusion therapies were the following: for IVT: infarct core (ADC < 620 µm2/s on diffusion MRI) < 70 ml, critically hypoperfused volume (Tmax > 6 s on perfusion MRI)/infarct core ratio > 1.2, mismatch volume (penumbra) > 10 ml (EXTEND study criteria); for EVT: core < 70 ml, ischaemia/core ratio > 1,8, penumbra > 15 ml (DEFUSE 3 study criteria).

In unknown onset strokes recognized within 4 h, IVT was also considered in the case of MRI DWI-FLAIR mismatch and infarct core < 70 ml (WAKE-UP study criteria). Wake-up strokes within 9 h from the midpoint of sleep with a core/perfusion mismatch were IVT candidates. In our analysis, wake-up strokes were grouped based on last seen or claimed well time: if within 4.5 h of presentation, they were assigned to the standard group; if not, to the extended group. All IVT patients were administered Alteplase 0.9 mg/kg (max 90 mg). IVT was always considered in patients with LVO before EVT [2]; however, it was not always performed because of an IVT contraindication like anticoagulation use or recent surgery.

Early EVT was indicated in intracranial ICA, MCA M1 and M2, ACA A1, PCA P1, and basilar occlusions, whereas late EVT was only considered in ICA, M1, and basilar occlusions. Late EVT indication was based on DEFUSE-3 criteria up to 24 h in anterior LVO. In basilar occlusions, late treatment was considered if NIHSS >  = 10 and there was no extensive brainstem damage on DWI-MRI [1].

EVT was provided by a team of experienced interventionalists, performing about 400 EVTs annually at the Department of Neurosurgery and Neurointervention at another site in a drip-and-ship model requiring secondary ambulance transportation*.* This institution is located 8 km away from the primary stroke centre resulting in net ambulance driving times between 10 and 20 min, depending on traffic conditions. The SARS-CoV-2 pandemic saturating ambulance service capacities partially overlapped with our study period [8], resulting in limited ambulance availability and increased secondary transport times. Postoperative care was provided at the Department of Neurosurgery and Neurointervention at a high-dependency unit.

The collected data consisted of demographic data (sex, age), stroke onset or last seen well time, admission time, NIHSS upon admission, imaging modality, door to imaging time (DIT), presence and location (anterior vs posterior) of large vessel occlusion (LVO), door-to-needle time (DNT), door-to-groin time (DGT), technical success of recanalization (TICI score), complication rate of EVT, and modified Rankin scale (mRS) at 90 days in treated patients. EVT complications were defined as artery perforation, dissection, subarachnoidal haemorrhage or symptomatic intracranial bleeding. The 3-month mRS was assessed via telephone call or in-person visit. The vital status (dead or alive) of patients lost to follow-up was checked from the national digital healthcare database. Independent functional outcome was defined as mRS ≤ 2.

DNT is defined as the time between a patient’s arrival at a healthcare facility (“door”) and the administration of an iv. thrombolytic agent (“needle”). DGT refers to the time between arrival at the first hospital site and the puncture time of the artery (“groin”) at the second site to perform the endovascular therapy.

This study was approved by the Semmelweis University Regional and Institutional Committee of Research Ethics (approval no. 123/2019) on October 19, 2019.

Patient consent was obtained for treatment in the extended thrombolysis time window (off-label use). For treatment in the standard time window and for data collection without treatment, consent was not obtained because the data were analysed anonymously. Data was accessed between the 1st and 15th of May, 2022, for research purposes. The first and last authors had information that could identify individual participants during data collection.

Statistical analysis was conducted between patient groups using either unpaired *t*-test, Mann–Whitney *U* test, Fisher’s exact test, or Chi-square test. Values are presented as mean ± standard deviation (SD) for age and as median, interquartile range (IQR) for NIHSS, mRS, and time metrics. For all analyses, an alpha value < 0.05 was considered significant. All analyses were performed using GraphPad Prism version 9.

## Results

During the 1-year study period, 777 confirmed ischaemic stroke patients were admitted to the ER within 24 h of symptom onset: 304 in the 0–4.5 h TW, an additional 82 in the 4.5–6 h TW, 149 in the 6–9 h TW, and 242 more patients were screened in the 9–24 h TW. A total of 252 (32.4%) had MRI during working hours (08–20 h); the others had CT-CTA.

### Comparing IVT in the standard and extended time window

Age, sex, and NIHSS upon admission were similar in the standard and the extended TW groups. A much higher proportion of patients were treated with IVT in the standard (~ 39%) than in the extended TW (~ 6%). In other words, to find one treatment eligible patient 2 had to be screened (number needed to screen: NNS = 2) in the early, whereas 9 in the late period. Clinical data and IVT outcomes in treated patients are presented in Table 1.

**Table 1:** All patients in IVT TW, in the indented section: clinical data of patients treated with IVT (0–9 h)

	Standard (0–4.5 h)	Extended (4.5–9 h)	*p* (statistics)
No of cases	304	231	-
Age mean (SD) years	67.5 (± 14.1)	68.5 (± 13.8)	0.28 (Mann–Whitney *U*)
Male %	51.6	55	0.81 (Chi-square)
NIHSS (median, IQR)	5 (2–13)	5 (2–8.25)	0.85 (Mann–Whitney *U*)
IVT	119 (39.1%)	14 (6.1%)	** < 0.001 (Chi-square)**
Age (mean, SD)	68.04 (± 1 1.9)	69.6 (± 14.8)	0.66 (Unpaired *t*)
Male %	55.4%	28.6%	0.08 (Fisher’s exact)
NIHSS (median, IQR)	7 (4–13)	8 (4–15)	0.61 (Mann–Whitney *U*)
mRS 90 days (median, IQR)	3 (1–5)	3 (1.75–6)	0.16 (Mann–Whitney *U*)
mRS 90 days ≤ 2% (nr/nr)	48.03% (49/102)	28.58% (4/14)	0.25 (Fisher’s exact)
Mortality rate	21%	35.71%	0.21 (Chi-square)
Number needed to screen (NNS)	2	9	-

The median (IQR) mRS at 90 days in the IVT group was 3 (1–5) in the standard and 3 (1.75–6) in the extended TW group (*p* = 0.16). Independent clinical outcome (mRS ≤ 2) at 90 days was seen in 49/102 (48.03%) early and 4/14 (28.58%) late-treated patients.

### Comparing EVT in the standard and extended time window

There were no differences in sex, initial NIHSS, and LVO rates between the standard and extended TW patients (Table 2*.*). Age in the late group was slightly higher. 27 patients had IVT before EVT in the early and 2 in the late TW. EVT was significantly more often performed among all patients screened in the standard TW (~ 9% vs 4%). Furthermore, a higher proportion of LVO patients had thrombectomy in the early compared to the late TW (~ 38% vs ~ 18%). Anterior circulation LVOs were predominant in both TWs (71/89 vs 61/82, *p* = 0.40). NNS was 10 in the early and 24 in the late TW. In treated patients, baseline clinical characteristics were balanced between the standard and extended TW groups. The recanalisation (TICI ≥ 2b) rate was equally high in early and late-treated patients (94% vs 93%). EVT-related complication rates were not statistically different (4/34 vs 5/15). Independent clinical outcome (mRS ≤ 2) at 90 days was seen in 38.4% in early and 33.3% in late-treated patients.

**Table 2:** All patients in EVT TW, in the indented section: clinical data of patients treated with EVT (0–24 h)

	Standard (0–6 h)	Extended (6–24 h)	*p* (statistics)
Number of cases	386	391	-
Age mean (SD) years	67.5 (± 14)	69.8 (± 13.8)	**0.017 (Mann–Whitney *U*)**
Male %	51.6%	51.9%	0.91 (Chi-square)
NIHSS (median, IQR)	5 (2–10)	4 (2–8)	0.92 (Mann–Whitney *U*)
LVO	89 (23.05%)	82 (20.9%)	0.48 (Chi-square)
EVT	34 (8.8%)	15 (3.8%)	**0.0044 (Chi-square)**
Age (mean, SD)	64.5 (± 11.9)	65.3 (± 16.4)	0.86 (unpaired *t*)
Male %	52.6%	53.3%	0.58 (Chi-square)
NIHSS (median, IQR)	13 (8–15.25)	12 (8–15)	0.9 (unpaired *t*)
LVO location anterior/total (%)	30/34 (88.2%)	11/15 (73.3%)	0.22 (Fisher’s exact)
TICI score ≥ 2b (%)	32/34 (94.1%)	14/15 (93.3%)	0.99 (Fisher’s exact)
Complications	4/34 (11.8%)	5/15 (33.3%)	0.109 (Fisher’s exact)
mRS 90 days (median, IQR)	3 (1.75–6)	6 (2–6)	0.43 (Mann–Whitney *U*)
mRS 90 days (mean, SD)	3.61 ± 2.1	4.2 ± 2.4	
mRS 90 days ≤ 2% (nr/nr)	38.4% (10/26)	33.3% (4/12)	0.99 (Fisher’s exact)
Mortality rate	26.4%	46.66%	0.07 (Chi-square)
EVT in LVO patients	34/89 (38.2%)	15/82 (18.2%)	**0.0009 (Chi-square)**
LVO location anterior/total (%)	71/89 (79.7%)	61/82 (74.4%)	0.40 (Chi-square)
NNS	10	24	-

### Comparing treatment times

We compared treatment times (median (IQR)) between imaging modalities (in the same TW) and between TWs (with the same imaging modality: MRI).

#### CT vs MRI in IVT

In the standard TW, out of the 119 IVT patients, 38 (31.9%) had MRI, and 81 (68%) had CT. Door-to-needle time (DNT) in patients who had MRI was slightly longer compared to CT in the standard TW: 69 (60–87.25) vs. 60 (47–82) min; Mann–Whitney *U* test: *p* = 0.0405.

#### Standard vs extended IVT with MRI

In the MRI thrombolysis group, 38 patients were treated in the standard and 13 in the extended TW. DNT in the extended group was significantly longer: 111 (74–161.5), vs 69 (60–87.25) min, Mann–Whitney *U* test: *p* = 0.002.

#### CT vs MRI in EVT

Of the 34 thrombectomy patients in the standard TW, 13 (38.2%) had MRI, and 21 (61.8%) had CT. In the standard TW, door-to-groin time (DGT) was numerically longer in patients who had MRI compared to CT although not statistically different: 210 (185–303) vs 150 (115–220) min, Mann–Whitney *U* test: *p* = 0.0566.

#### Standard vs extended EVT with MRI

DGT using MRI in the standard TW was similar to the extended TW (210 (185–303) vs 229 (161–241) min, *p* = 0.99, unpaired *t*-test.

### Comparing door to imaging times (DIT)

We compared door-to-imaging times (DIT, median (IQR)) between imaging modalities (in the same TW) and between TWs (with the same imaging modality: MRI) in treated patients; and also in all screened patients regardless of whether they eventually received treatment.

#### CT vs MR in treated patients

In the standard TW in the case of IVT-treated patients, DIT was numerically shorter for CT although not statistically significant: 28.5 (20–49.75) vs 33.5 (24.5–50.25) min, Mann–Whitney *U* test: *p* = 0.28.

In the standard TW EVT-treated patients, DIT with CT was significantly shorter than MRI, 20 (15–30) vs 33.50 (26.75–56.25) min, Mann–Whitney *U* test: *p* = 0.002.

#### CT vs MR in all screened patients

Among all patients screened within 24 h (the entire study population), there was no significant difference in DIT CT vs MRI: 73.50 (34–177) vs 61.00 (38–125) min, Mann–Whitney *U* test: *p* = 0.12.

Among all patients screened within 4.5 h, there was no significant difference in DIT CT vs MRI: 42 (24–102I vs 50 (31–88) min, Mann–Whitney *U* test: *p* = 0.39.

#### Standard vs extended time window in all MRI-screened patients

Among all patients screened with MRI DIT differed significantly between early and late time windows: for 0–4.5 vs 4.5–9 h TW (50 (31–88) vs 62.5 (37.5–142.8) min, Mann–Whitney *U* test: *p* = 0.039) and for 0–6 vs 6–24 h TW (51 (32.5–102.5) vs 80.5 (42.5–160.8) min, Mann–Whitney *U* test: *p* = 0.0022).

## Discussion

Implementing the 2021 ESO guidelines in our patient management and extending the time window for possible reperfusion treatment from 6 to 24 h resulted in a 102% increase in the number of patients (from 386 in the standard TWs in this study to 777 within 24 h) requiring emergent screening within 24 h of symptom onset. Using MRI as routine first-choice imaging in stroke started at the beginning of the study period. Previously MRI was only considered for selected patients such as wake-up or unknown TW strokes, posterior fossa strokes and possible stroke mimics.

### IVT

With the extension of the TW from 4.5 to 9 h, there was a 27% increase in potential IVT candidates compared to the standard TW alone and an 11.7% increase in the number of actual IVTs performed in the standard and extended TW combined compared to the standard alone. The overall rate of IVT in the entire cohort was 17.1%—comparable to previous data from our centre (11.5% in 2017, 18.1% in 2018) [9]. Another centre from Budapest screened 408 patients within a 6-month period in 2023 and had an IVT rate of 15% (only CT-based standard TW treatment) [10].

Using MRI as first-choice imaging instead of CT significantly increased the DNT in the standard TW (69 vs 60 min). DIT was also numerically longer for MRI than CT, although not significantly. The significant difference in DNT can be explained by the longer transportation and interpretation time required for MRI. Treatment times as well as imaging times were much longer in late than early IVT patients undergoing MRI (DNT 111 vs 69 min, DIT 62.5 vs 50 min). The important prolongation of DNT in the extended TW can be explained by the longer MRI protocol, the need for the relatively time-consuming core-penumbra calculations for decision making and perhaps a lower sense of urgency in this patient group.

With similar age and NIHSS at admission, the median mRS at 90 days was similar in the two IVT TWs. Functional independence was numerically more common in the standard TW although not significant. This can be explained by the low number of patients in the extended TW group, but may also suggest that earlier treatment still results in better stroke outcomes; however, there is definitely a noteworthy group of IVT candidates in the extended TW who benefit from IVT compared to a control group [11, 12]. An almost fivefold increase in NNS in the extended TW for IVT compared to the standard TW highlights the increasing clinical burden and decreasing efficiency of screening these late patients.

There have been randomized clinical trials on administering 0.25 mg/kg tenecteplase beyond the standard IVT time window [13–18] before thrombectomy or in wake-up strokes recognized within 4.5 h since awakening, and the results seem promising. However, currently, there is not enough evidence to provide thrombolysis beyond 9 h routinely and without advanced imaging; and the global unavailability of tenecteplase limits its current use.

### EVT

By extending the time window from 6 to 24 h, we screened twice as many patients for EVT resulting in an 89% increase in potentially treatable LVO patients, and a 44% increase in actual EVTs performed. NNS to perform an EVT in extended TW was 2.5-fold higher than in the standard TW, which still compares favourably to the extended TW IVT group. The overall rate of EVT in the entire cohort was 6.3%—a meaningful increase compared to previous data from our centre (2.8% in 2017, 4.8% in 2018) [9].

DGT with MRI was not significantly longer than with CT in the standard TW and was also similar in early and late EVT with MRIs. However, DIT was significantly longer in the standard TW EVT patients with MRI than CT (33.5 vs 20 min). It seems that in more severe LVO stroke patients where the clinical case is more straightforward and urging, CT does provide a significantly faster imaging solution. The lack of significant difference in DGT between imaging modalities is due to the dilution of the time benefit of CT by the much longer secondary transport time in the drip-and-ship model. DIT was significantly shorter in early than late TW among all patients screened with MRI (51 vs 80.5 min) probably due to a higher sense of urgency.

With the guideline-based approach and a well-defined patient pathway, our results improved compared to a previous study from our group where we used CTA screening before MRI to select late time-window patients for EVT [7]. With similar age and NIHSS at admission, functional outcomes of treatments in the standard and extended TWs were comparable at 90 days. Similar to other studies, there was an overall favourable functional outcome (mRS ≤ 2) of about 30–40% in both the standard and extended EVT TWs [19–22].

In light of the new large core EVT trials [23–25] and their meta-analysis [26], there is growing evidence that EVT can be offered to anterior LVO patients for up to 24 h based only on CT and CTA. The overwhelming efficacy of EVT in LVO strokes questions the need for advanced imaging in severe stroke cases. These studies also point out that current guideline-based selection criteria for EVT [1] may be too restrictive, especially in the 6–24 h time window [5]. We have already changed our local protocol to reflect these new pieces of evidence which will certainly further increase the rate of EVT and blur the distinction between early and late LVO patients within 24 h.

The prolonged treatment times associated with the use of MRI in our centre are mainly due to logistical reasons and suggest that using advanced imaging in straightforward early patients and suspected LVO patients might be excessive. In line with the “time is brain” concept, in uncomplicated early and probable LVO cases, CT and CTA carry enough information for acute decision-making, especially if aided by artificial intelligence-based automatic software [9, 27]. Penumbral advanced imaging is still useful for patient selection for extended TW IVT (non-LVO patients), for detection of distal branch occlusion and for differential diagnosis in possible stroke mimics (other medical conditions mimicking stroke) and chameleon strokes (atypical or misleading stroke symptoms resembling other medical conditions).

## Limitations

The most important limitation of our study from a clinical point of view is the limited availability of MRI. There were 231 patients who arrived within 4.5–9 h of stroke onset for whom only CT scan was available without perfusion imaging; therefore they were not considered for thrombolysis.

A few patients were lost to follow-up (with only their vital status available), mostly in the standard time window groups; therefore it is unlikely to change our favourable results for late-treated patients. This can be explained by the complex patient pathways in the Budapest region: patients residing outside our catchment area who are taken acutely to our centre because of the geographical proximity of their place of discovery are later managed by another healthcare provider.

The single-centre nature of this study limits generalizability, but it is of note that this is the first centre in Hungary to perform extended time window reperfusion therapies and use MRI routinely in the hyperacute setting. Previous data from Hungary regarding acute stroke care is available in relation to AIS hospitalization and mortality rates compared to other European countries [28], acute stroke onset to imaging and treatment time in the COVID-19 pandemic era in comparison to the previous year [29], and AIS treatment times and percentages within the recommended standard time window in another Budapest stroke-centre using CT and CTA [10]; but none regarding extended time window reperfusion therapies with time metrics or any experience with acute stroke MRI.

As stated above, our results for extended TW EVT are less relevant in light of the recent large core clinical trials.

Another limitation is the low number of treated patients in the extended TWs, especially for IVT. For example, functional independence was higher, yet not statistically significant in the standard IVT group compared to the extended (48% vs 28.6%) group mainly because of the low number of patients in the extended group. However, we will not continue our study with the same criteria as we have recently changed our local protocol.

In an era when acute stroke treatment practice changes rapidly due to frequently emerging new evidence, our results may seem slightly outdated; however, they still reflect practice based on international guidelines (AHA and ESO) currently in force. As mentioned above, we have also changed our local protocols to better reflect new evidence and provide treatment to a wider population of patients.

## Conclusions

Even with limited MRI availability, the extension of the TWs for acute reperfusion therapies resulted in a significant increase in treatment numbers with comparable clinical outcomes in early and late-treated patients. This increase in treatment rates was moderate for IVT and more remarkable for EVT and came at the price of a significantly increased burden of acute clinical and advanced imaging screening.

Arrival to imaging and arrival to treatment times were shorter with CT than with MRI. Advanced imaging is very useful in selecting late or unknown-onset patients for extended TW IVT and in diagnostic uncertainties. However, for early straightforward patients and those with suspected LVO, simple CT and CTA may be enough for decision-making.

## Data Availability

All data is available upon request from the corresponding author.

## Abbreviations

ACA: Anterior cerebral artery
ADC: Apparent diffusion coefficient
ASPECTS: Alberta stroke program early CT score
CT: Computed tomography
CTA: Computed tomography angiography
CTP: Computed tomography perfusion
DGT: Door-to-groin time
DNT: Door-to-needle time
DWI: Diffusion-weighted imaging
EVT: Endovascular therapy
GRE: Gradient echo
FLAIR: Fluid-attenuated inversion recovery
ICA: Internal carotid artery
IRQ: Interquartile range
IVT: Intravenous thrombolysis
LVO: Large vessel occlusion
MCA: Middle cerebral artery
MRI: Magnetic resonance imaging
MRP: Magnetic resonance perfusion
mRS: Modified Rankin Scale
NIHSS: National Institutes of Health Stroke Scale
NNS: Number needed to screen
PCA: Posterior cerebral artery
PWI: Perfusion-weighted imaging
SD: Standard deviation
SWI: Susceptibility-weighted imaging
TW: Time window
